# Outcomes of Surgical Resection of Primary Lung Cancer After Pancreatic Cancer

**DOI:** 10.7759/cureus.73689

**Published:** 2024-11-14

**Authors:** Yudai Miyashita, Naoko Ose, Jiro Okami, Koji Takami, Yasushi Sakamaki, Naoki Ikeda, Ken Kodama, Toshiteru Tokunaga, Yasushi Shintani

**Affiliations:** 1 Department of General Thoracic Surgery, Osaka University Graduate School of Medicine, Suita, JPN; 2 Department of Thoracic Surgery, Osaka International Cancer Institute, Osaka, JPN; 3 Department of Thoracic Surgery, National Hospital Organization Osaka National Hospital, Osaka, JPN; 4 Department of Chest Surgery, Osaka Police Hospital, Osaka, JPN; 5 Department of Thoracic Surgery, Sakai City Medical Center, Sakai, JPN; 6 Department of Thoracic Surgery, Yao Municipal Hospital, Yao, JPN; 7 Department of General Thoracic Surgery, National Hospital Organization Kinki-Chuo Chest Medical Center, Sakai, JPN

**Keywords:** lung lobectomy, lung metastasis, pancreatic cancer, primary lung cancer, recurrent

## Abstract

Objective: This study aimed to elucidate the therapeutic significance of lung resection for primary lung cancer after pancreatic cancer surgery in contemporary cases.

Methods: This retrospective cohort study included patients who had lung nodules and performed pulmonary resection after pancreatic cancer surgery at seven hospitals affiliated with the Thoracic Surgery Study Group of Osaka University between January 2009 and December 2021. Patients in which surgery was performed for biopsy purposes, those with a history of other cancers with potential for lung metastasis, patients who did not give their consent for enrollment, and patients determined to be ineligible by the attending physician were excluded from the study.

Results: A demographic analysis revealed that 17 patients were eligible for inclusion. Pathological diagnoses were established by institutional pathologists and occasionally aided by immunostaining and genetic testing. A survival analysis revealed a 3-year survival rate of 61.9% and a 5-year survival rate of 54.2% after lung resection. Subgroup analyses highlighted the impact of the interval between pancreatic cancer surgery and lung nodule detection, tumor diameter, and procedure on survival outcomes.

Conclusions: This study underscores the therapeutic implications of lung resection for primary lung cancer following surgery for pancreatic cancer. Despite the challenges in preoperative diagnosis and treatment decisions, surgical intervention demonstrates promise, especially in select cases. Further research is needed to determine the best therapeutic strategies for this group.

## Introduction

Pancreatic cancer is one of the most formidable malignancies, characterized by a survival time of only 12.6 months (median) and a poor 5-year survival (7%) [1]. This grim outcome is compounded by the fact that many cases are diagnosed at a late stage and the development of recurrent disease after pancreatic cancer surgery, as evidenced by studies such as those conducted by Egawa and Javadi [2,3]. Moreover, despite surgical intervention, the 3-year disease-specific survival rate (DSR) remains low at 27% [4].

Nevertheless, due to advancements in multidisciplinary treatment modalities and early pancreatic cancer detection, there has been a noticeable improvement in the prognosis of pancreatic cancer, which fosters optimism [2,5]. With the anticipated rise in long-term survivors, we are seeing an increased incidence of lung nodules following pancreatic cancer surgery.

It is crucial to distinguish lung nodules following pancreatic cancer surgery from primary lung cancer or metastatic lung tumors. Typically, the imaging findings of metastatic lung tumors exhibit relatively distinct tumor shadow margins and round or oval nodular shadows. However, they may occasionally present with cavities, calcifications, pleural insertions, and standard features in primary lung cancer, which makes it challenging to differentiate based solely on imaging findings [6]. Thus, obtaining a tissue biopsy prior to surgery is advisable. Nevertheless, obtaining preoperative histological biopsy specimens for the diagnosis of postoperative lung nodules originating from pancreatic cancer is challenging, with cases often detected when tumors are small, with peripheral lesions being prevalent.

Furthermore, distinguishing pulmonary metastases of pancreatic cancer from primary lung adenocarcinoma is an intricate process, and bronchoscopic specimens often fail to yield a definitive diagnosis [7]. Consequently, combined diagnostic and therapeutic lung resection may be necessary, as it has the potential to reveal primary lung cancer after surgery. The only comprehensive report on surgical resection of primary lung cancer after post-pancreatic cancer surgery is a 13-case study by Robinson et al. in 2016. The study demonstrated relatively favorable prognoses. However, definitive conclusions regarding surgical resection remain elusive due to the limited number of reported cases [8].

The present study aimed to elucidate the therapeutic relevance of lung resection for primary lung cancer after pancreatic cancer surgery in the current era.

## Materials and methods

Patients

We conducted a retrospective cohort study of all patients who underwent pulmonary resection for primary lung cancer with a history of surgery for pancreatic cancer at seven institutions affiliated with the Thoracic Surgery Study Group of Osaka (TSSGO) University between January 2009 and December 2021. Cases involving multiple lung surgeries to achieve complete resection were also included. Cases in which surgery was performed for biopsy purposes, cases with a history of other cancers, and cases in which enrollment was refused were excluded. In this retrospective study, no interventions were performed, and patient consent was obtained through an opt-out process. This means that patients were informed about the study and were given the opportunity to decline participation. Those who did not opt-out were considered to have consented to the use of their medical records for research purposes.

Pathological diagnosis

Pathologists diagnosed the primary lung cancer at each hospital. Immunostaining for Thyroid Transcription Factor-1 (TTF-1) and Napsin A and genetic testing were conducted at the pathologist's discretion. The diagnosis of primary lung cancer was established clinically. However, pathological findings could not conclusively diagnose pulmonary metastasis originating from pancreatic cancer or primary lung cancer, mainly due to invasive mucinous adenocarcinoma (IMA).

Evaluation criteria

The primary endpoint was overall survival (OS), and the secondary endpoint was the identification of prognostic factors. The study protocol received approval from the Ethics Committee of Osaka University Hospital (control number 20160-2) and the institutional review boards of the participating institutes.

Methods

Nodules in the lung were diagnosed based on chest computed tomography (CT) findings. The primary pancreatic tumors were diagnosed pathologically and were treated surgically in all patients prior to lung resection. Pathologists evaluated tissues of resected pulmonary nodules histologically, and all patients were diagnosed with primary lung cancer. We collected the clinical information from the participating institutes' medical records.

After considering the patient's general condition and the disease's state, the general surgeon-in-charge determined the indications for pre and postoperative chemotherapy or radiotherapy. The type of surgical approach and resection were chosen based on the size and location of the pulmonary nodules. Follow-up was generally conducted on chest radiography or chest CT, blood chemistry, and physical examination, performed every 6-12 months after the initial lung resection. Clinical and postoperative staging were classified according to the 7th edition of the tumor, node and metastasis (TNM) staging system for lung and pancreatic cancer [9,10].

The interval from pancreatic cancer surgery to primary lung cancer discovery (IPL) was defined as the time from the treatment of the primary pancreatic tumor to the detection of pulmonary nodules. For patients whose primary pancreatic tumor and lung nodules were diagnosed simultaneously at the initial presentation, this interval was recorded as zero. The disease-free interval (DFI) was defined as the time from treatment for primary lung cancer to the identification of primary lung cancer recurrence. In cases where recurrence was suspected, if a biopsy was feasible, pathological findings were used to differentiate whether the recurrence originated from lung or pancreatic cancer. When a pathological diagnosis was unavailable, we clinically determined the origin of the recurrence. OS was defined as the duration from the date of lung resection to death or the last follow-up for patients who were still alive. In this study, the follow-up period was measured from the date of pulmonary resection to either the date of death or the most recent follow-up. The follow-up periods at the 75th quartile, median, and 25th quartile were 75, 33, and 27 months, respectively (range: 15-129 months).

Statistical analysis

The Kaplan-Meier method was used to illustrate the survival curve and calculate the survival rate. Statistical differences between survival curves were assessed using the log-rank test, with a p-value of <0.05 indicating statistical significance. Data are presented as the median±standard deviation. All statistical analyses were performed by the author (YM) using JMP Pro 17 software (SAS Institute Inc., Cary, NC, USA).

## Results

Demographics

 During the study period, 10,029 surgeries for primary lung cancer were performed in the TSSGO group, and 17 patients (0.17%) were enrolled in the study. The 17 patients' characteristics are presented in Tables 1-2. The median patient age was 71 years. The median body mass index (BMI) was 20.7. The median forced expiratory volume in one second (FEV1) was 2.35L. The median carbohydrate antigen 19-9 (CA19-9) and carcinoembryonic antigen (CEA) were 13.5 U/mL and 3.8 ng/mL, respectively.

**Table 1:** Patients’ characteristics and image findings BMI: body mass index; PS: performance status; FEV1: forced expiratory volume in one second; CA19-9: carbohydrate antigen 19-9; CEA: carcinoembryonic antigen; PC: pancreatic cancer; PD: pancreaticoduodenectomy; DP: distal pancreatectomy; UNK: unknown; HF: histological findings; IPMN: intraductal papillary mucinous neoplasm; IDC: invasive ductal carcinoma; MCN: mucinous cystic neoplasm; IPL: interval from PC surgery to PLC discovery; DPS: distance from pleural surface; RUL: right upper lobe; RML: right middle lobe; RLL: right lower lobe; LUL: left upper lobe; LLL: left lower lobe, meta; CTR: consolidation tumor ratio; PLC: primary lung cancer.

No.	Age/ Gender	BMI	PS	FEV1 (L)	CA19-9 [U/ml]	CEA (ng/ml)	Stage (PC)	Surgery (PC)	HF (PC)	IPL (mo)	Size (mm)	number of nodules	Localization	Side	Border	Shape	Cavity	Calcification	CTR	preoperative diagnosis
1	58/M	19.8	0	3	0	5	0	PD	IPMN	0	15	1	RUL	one	irregular	other	-	-	1	PLC
2	71/F	18.1	0	1.8	309	39.8	3	PD	IDC	0	11	2	LLL	one	regular	circle	-	-	1	PLC
3	61/M	16.1	0	2.4	8	2.7	2a	DP	IDC	36	3	2	RLL	both	regular	circle	-	-	0	UNK
4	77/M	22.3	1	2	-	58.2	2b	PD	IDC	37	21	1	LLL	one	regular	circle	-	-	1	PLC
5	58/M	22.2	0	2.4	24	2	3	PD	IDC	38	27	1	RLL	one	irregular	other	-	-	1	UNK
6	69/F	22.2	0	2	4	2.4	3	DP	IDC	40	22	1	LLL	one	irregular	other	-	-	1	PLC
7	75/M	22.8	0	2.5	95	3.8	3	PD	IDC	48	25	1	LUL	one	irregular	other	-	-	1	meta
8	77/F	16.3	0	2.1	12	3.9	1a	PD	IDC	53	14	3	RLL	one	irregular	other	+	-	1	meta
9	65/M	20.7	0	3.2	-	3.7	1a	DP	IDC	96	12	1	RUL	one	irregular	circle	-	-	1	PLC
10	70/F	16.6	0	2.3	15	6.4	2a	DP	IDC	10	35	1	LUL	one	irregular	circle	+	+	1	PLC
11	53/M	25.7	1	2.4	101	6.5	2a	DP	IDC	11	20	1	RUL	one	regular	circle	-	-	1	PLC
12	78/M	18	0	2.5	19	4.5	2b	DP	IDC	15	35	1	RUL	one	irregular	other	+	-	0.6	PLC
13	79/F	23.7	0	1.7	8	4	1a	DP	MCN	0	18	1	RUL	one	irregular	other	-	-	1	PLC
14	81/M	18.7	1	1.3	8	3.8	1a	DP	IPMN	15	9	1	LLL	one	irregular	other	-	-	1	PLC
15	62/M	20.7	0	3	-	2.4	2a	DP	IDC	48	8	1	RML	one	regular	circle	-	-	1	PLC
16	74/F	28.8	0	1.9	-	1.6	UNK	UNK	UNK	168	6	1	RML	one	irregular	other	-	-	1	UNK
17	72/M	20.8	0	2.4	-	1.6	UNK	UNK	UNK	172	18	1	RUL	one	regular	circle	-	-	0	PLC

**Table 2:** Treatment and survival VATS: video-assisted thoracic surgery; RATS: robot-assisted thoracic surgery; HF: histological findings; LCC: large cell carcinoma; ACA: acinar adenocarcinoma; IMA: invasive mucinous adenocarcinoma; SCC: small cell carcinoma; sq: squamous cell carcinoma; PAP: papillary adenocarcinoma; mixed: mixed subtype; AIS: adenocarcinoma in situ; PLC: primary lung cancer; CT: chemotherapy; RT: radiotherapy; CRT: chemoradiotherapy; DFI: disease free interval; PC: pancreatic cancer; LN: lymph node; TPR: treatment for postoperative recurrence; BSC: best supportive care; OS: overall survival; DOD: die of disease; AWD: alive with disease; NED: no evidence of disease; UNK: unknown.

No.	Approach	Surgery	HF(PLC)	Stage (PLC)	Adjuvant therapy	DFI(mo)	Recurrence	Recurrence organ	TPR	OS	Current status
1	VATS	Lob	LCC	1b	CT	8	PLC	brain	CRT	26	AWD
2	VATS	Lob	ACA	2b	none	1	PLC	lung	surgery	28	DOD
3	VATS	Wed	IMA	4	surgery	34	PLC	LN	CT	88	AWD
4	RATS	Lob	SCC	1a	CT	13	PLC	lung, liver, bone	CT	19	DOD
5	Thoracotomy	Lob	sq	2a	none	22	PLC	lung	CT	129	DOD
6	Thoracotomy	Lob	IMA	2a	CT	11	PLC	bone, brain, meninges	BSC	15	DOD
7	VATS	Lob	IMA	1b	none	16	PLC	pleura	RT	32	AWD
8	Thoracotomy	Seg	PAP	1a	none	70	PLC	pleura	BSC	80	AWD
9	Thoracotomy	Lob	IMA	1a	none	9	PLC	LN	CRT	33	DOD
10	VATS	Lob	PAP	1b	none	-	PC	lung	surgery	53	DOD
11	Thoracotomy	Lob	sq	2a	CT	-	PC	pancreas	CT	30	DOD
12	VATS	Lob	mixed	1a	none	-	PC	peritoneum	CT	21	DOD
13	VATS	Seg	ACA	1a	none	-	-	-	-	30	UNK
14	VATS	Wed	sq	1a	none		-	-	-	70	NED
15	VATS	Lob	IMA	1a	none	-	-	-	-	63	NED
16	Thoracotomy	Lob	PAP	1a	none	-	-	-	-	60	NED
17	VATS	Lob	AIS	0	none	-	-	-	-	87	NED

Pancreatic cancer-related factors

The pancreatic cancer stages were 0, I, II, and III as 1, 4, 6, and 4 patients, respectively. Pancreaticoduodenectomy (PD) was selected for pancreatic cancer in six cases and other techniques in nine cases. The histopathological types of pancreatic cancer included invasive ductal carcinoma, intraductal papillary mucinous neoplasm (IPMN), and other or unknown types in 12, 2, and 3 patients, respectively. The interval from pancreatic cancer treatment to lung cancer discovery (IPL) was 37 months (range: 0-172 months).

Primary lung cancer-related factors

The median diameter of lung tumors was 18 mm. The median number of lung nodules was one, and solitary primary lung cancer was in 14 cases. The border was irregular in 11 and regular in 6. The shape was a circle in 8 (47.1%) and the other in 9 (52.9%). Ground-glass opacity nodules (GGNs) and cavity lesions were in 3 each. Calcification was observed in one case. The median consolidation tumor ratio was 1.0 (range: 0-1.0). Video-assisted thoracic surgery was a commonly used surgical approach. Wedge resection, segmentectomy, and lobectomy were present in 2 (11.8%), 2 (11.8%), and 13 (76.5%) cases, respectively. Lymph node dissection was performed in 13 cases. Complete resection was performed in 16 cases. In one case, complete resection was not achievable due to the presence of multiple lung nodules, and wedge resection of a pulmonary nodule in the contralateral lung was performed a few months later.

The preoperative diagnosis and surgical procedure

Out of the 17 patients, 4 (23.5%) were preoperatively diagnosed with primary lung cancer through histological examination, 8 (47.05%) were diagnosed with primary lung cancer through CT scan, 2 (11.7%) patients were suspected of having metastatic lung cancer based on CT scans. In the remaining 3 (17.6%) cases, the distinction between primary lung cancer and metastatic lung tumor was not made before surgery. Of the 12 (70.5%) patients with a preoperative diagnosis of primary lung cancer, 9 (75%) underwent lobectomy, and 3 (25%) underwent palliative sublobar resection. Of the 2 (11.7%) patients with a preoperative diagnosis of metastatic lung cancer, 1 (50%) had multiple nodules within the same lung lobe and underwent lobectomy, whereas the other had a single nodule and underwent sublobar resection. Of the 3 patients with no preoperative diagnosis, 2 (66.6%) were diagnosed with primary lung cancer based on intraoperative rapid pathology and underwent lobectomy, and 1 (33.3%) underwent lobectomy based on the tumor’s location.

Postoperative details

Postoperative details are shown in Table 2. The pathological findings of the lung tumors were as follows: adenocarcinoma (11, 64.7%), squamous cell carcinoma (3, 17.6%), small cell carcinoma, carcinoma in situ, and large cell carcinoma (1, 5.8%) each. Of the 11 adenocarcinomas, 5 (45%) were diagnosed as IMA, 3 (27.7%) as papillary, 2 (18.8%) as acinar, and 1 (9.09%) as mixed. The lung cancer stages were as follows: stage 0 (n=1), stage I (n=11), stage II (n=4), and stage IV (n=1). Throughout the observational interval, 9 (52.9%) patients experienced a recurrence of primary lung cancer, while 3 (17.6%) experienced a recurrence of pancreatic cancer. 8 (47.05%) patients died during the follow-up period.

Review of recurrent cases

Among the 12 individuals who experienced relapse, 9 (75%) manifested a relapse of primary lung cancer, while 3 (25%) experienced a recurrence of pancreatic cancer. Within the subgroup of 9 patients with recurrent primary lung cancer, 5 (55.5%) presented with stage I lung cancer, and 3 (33.3%) presented stage II disease, constituting a cohort characterized by relatively lower disease stages. Among the 6 patients, excluding cases classified as stage 1a, 2 (33.3%) had undergone postoperative chemotherapy after lung resection. The median DFI score was 13 months. 3 (50%) patients experienced recurrence of pancreatic cancer after surgical resection for primary lung cancer, with recurrence manifesting between 16 and 36 months after the initial pancreatic cancer surgery.

Survival analysis

**Figure 1:**
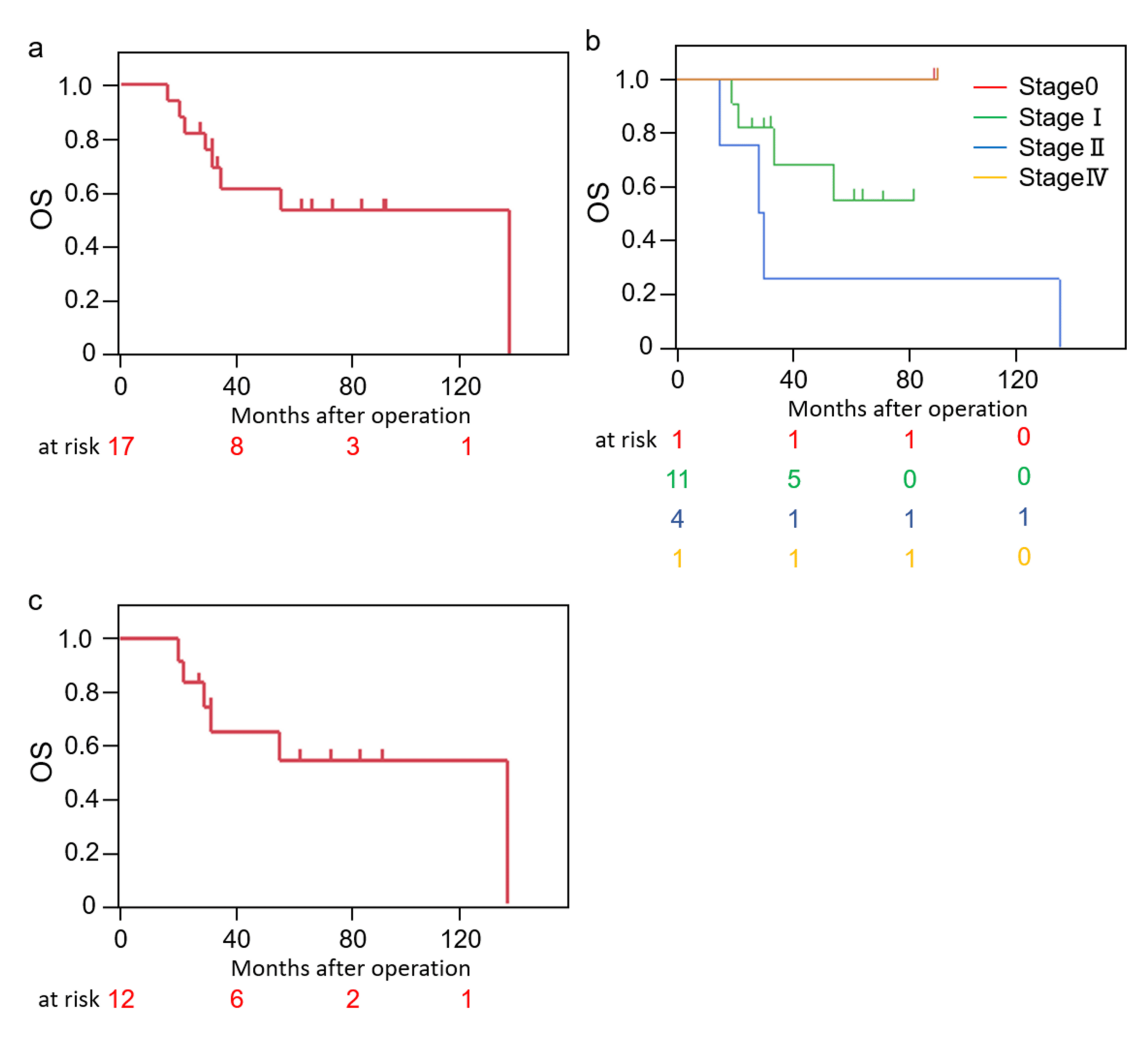
Overall survival a: overall survival; b: overall survival according to stage, c: overall survival, with the exclusion of cases of IMA; OS: overall survival; IMA: invasive mucinous adenocarcinoma.

A survival analysis was subsequently performed for 17 patients with resection of primary lung cancer (Figure 1a). The three and five-year survival rates after lung resection were 61.9% and 54.2%, respectively. Figure 1b shows the OS for each stage. The 5-year survival rates for each stage were as follows: stage 0, 100%; stage I, 54.6%; stage II, 25%; and stage IV, 100%. 12 cases, excluding those involving the IMA, amenable to the pathological diagnosis of primary lung cancer, were also investigated. The three and five-year survival rates of the 12 patients were 64.8% and 54.0%, respectively, and the results were similar to survival in a total of 17 cases (median follow-up periods, 41.5 months) (Figure 1c). 

Predictors of OS

**Figure 2:**
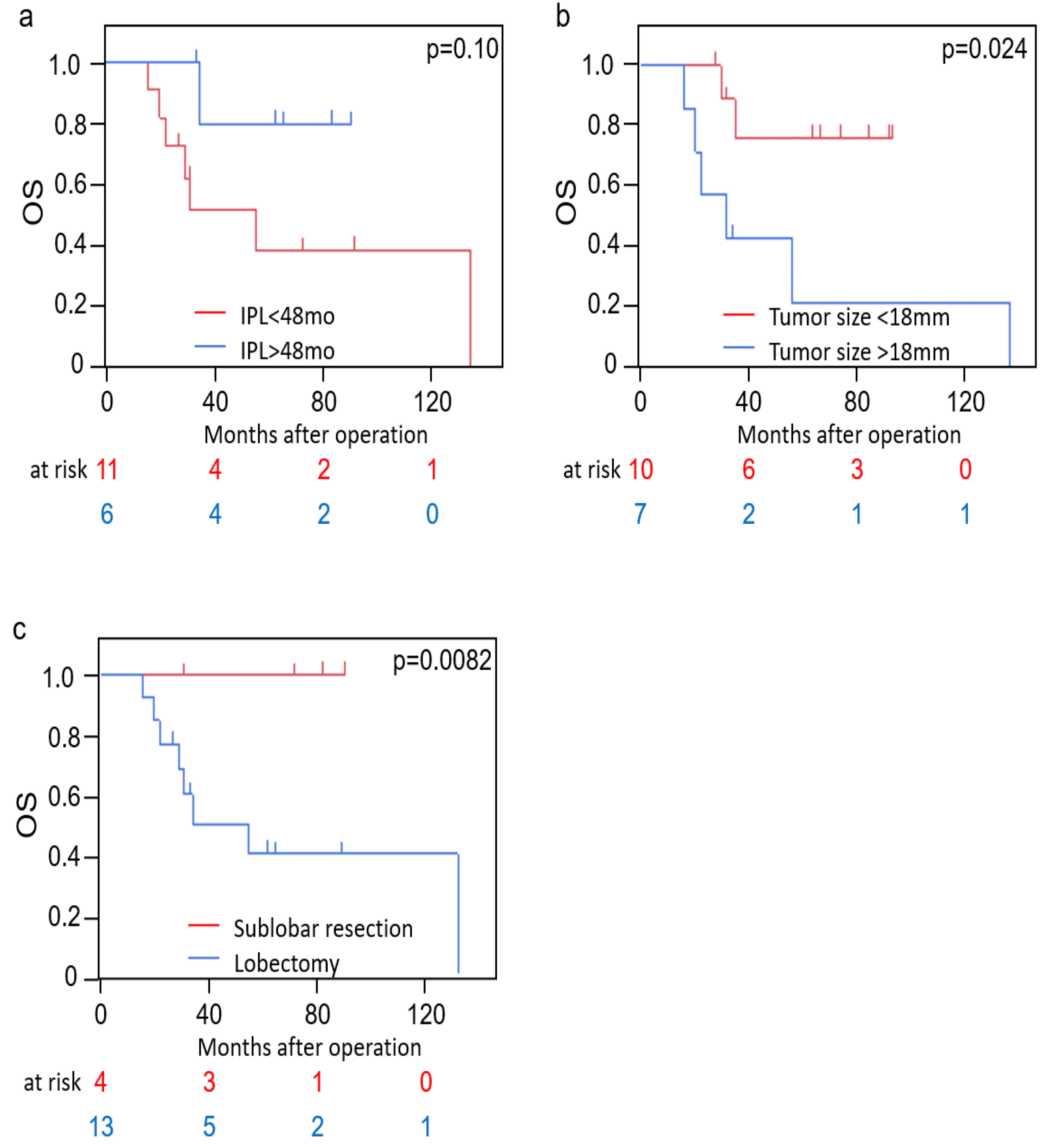
Overall survival according to the groups. a: overall survival according to the IPL; b: overall survival according to the tumor size; c: overall survival according to the surgery; IPL: interval from pancreatic cancer surgery to primary lung cancer discovery.

OS was assessed across all cases. Several factors were considered as risk factors for OS. The cut-off values were determined using the median or ROC curve. Among the 17 patients, those with an IPL of more than 48 months tended to have better OS compared to those with an IPL of less than 48 months (p=0.10). The three and five-year survival rates were 80.0% vs. 51.9% and 80.0% vs. 40.0%, respectively (Figure 2a). 10 of the 17 patients with a tumor size of <18 mm had better OS than those with a tumor size of >18 mm (p=0.024), with three and five-year survival rates of 76.2 vs. 42.9% and 76.2 vs. 21.4%, respectively (Figure 2b). 4 of 17 patients who underwent sublobar resection had better OS than those who underwent lobectomy (p=0.0082), with three and five-year survival rates of 100 vs. 49.9% and 100 vs. 39.9%, respectively (Figure 2c).

## Discussion

We present patient demographics and survival outcomes after pancreatic cancer surgery in patients with primary lung cancer. Travis et al. reported 5-year survival rates of 91-93% for stage I primary lung cancer and 85-86% for stage II primary lung cancer [11]. In contrast, our study revealed a less favorable prognosis, with survival rates of 54.6% and 25% in patients with stage I and II disease, respectively. Additionally, we investigated potential prognostic factors and identified IPL, tumor diameter, and surgical technique as significant determinants of the prognosis.

Ko et al. conducted a study involving 166 cases of primary lung cancer with antecedent malignancies and reported a 5-year survival rate of 72.9%. The corresponding 5-year survival rates for 28 patients with gastrointestinal tract malignancies and 8 patients with hepatocellular carcinoma were 43.1% and 46.7%, respectively. Moreover, the survival rates were notably higher for breast cancers (n=71; 76.7%), genitourinary tract malignancies (n=20; 86.9%), and thyroid cancers (n=11; 100%), indicating that gastrointestinal tract and liver cancers were associated with a less favorable prognosis [12]. Lim et al. reported a 5-year survival rate of 34.4% in 80 cases of iatrogenic secondary lung cancer after ovarian cancer and a 5-year survival rate of 40.1% in lung cancer following colon cancer, which is inferior to the survival rates observed in patients with lung cancer after thyroid cancer (82.6%) and breast cancer (70.5%) [13]. Robinson et al. provided the only available data on primary lung cancer post pancreatic cancer surgery, reporting a 5-year survival rate of 60.1% and a median survival time of 78 months among 13 cases [8]. In our investigation, the overall 5-year survival rate was in line with that reported by Robinson et al. and with the rates observed in other gastrointestinal malignancies.

Lung nodules following pancreatic cancer surgery often prompt inquiry into metastatic or primary lung cancers. In this investigation, we compiled data on imaging observations and tumor markers. In comparison to the 35 cases of metastatic lung tumors after pancreatic cancer surgery documented by our team in 2023, we found no noteworthy disparities in CT imaging findings. Similarly, no disparities were evident in the median tumor marker levels (CEA 3.8 ng/mL vs. 4.1 ng/mL, CA19-9 13.5 U/mL vs. 19.4 U/mL) [14]. As previously noted, achieving a preoperative diagnosis based on imaging findings and tumor markers appears to be challenging. Conversely, four cases diagnosed with squamous cell carcinoma via bronchoscopy received a preoperative diagnosis of primary lung cancer. However, instances have been reported in which the diagnosis of lung metastasis from pancreatic cancer or primary lung cancer was not attained when the bronchoscopy specimen was diagnosed as adenocarcinoma [7]. Hence, preoperative bronchoscopy may not be necessary.

In the present study, the duration of pancreatectomy showed positive associations with the identification of lung nodules and an improved prognosis. Similarly, in our analysis of metastatic lung tumors, the interval from pancreatectomy to detection of lung nodules emerged as a prognostic factor [15]. Among the nine instances of recurrence after primary lung cancer resection, three were pulmonary recurrences. Regrettably, only one of these cases underwent a tissue biopsy, while the remaining two cases potentially involved metastatic lung tumors. Conducting a biopsy may facilitate more tailored therapeutic interventions for carcinomas. Furthermore, the discrimination between primary lung cancer and metastatic lung tumors based on pathological findings remains elusive, particularly with regard to IMA. Although no prognostic variance was noted between IMA and the other subtypes in this study, the potential presence of mixed metastatic lung tumors within the case group could not be discounted.

In this study, lobectomy emerged as an adverse prognostic indicator. This outcome could be attributed to the considerable tumor size, which necessitates lobectomy in some patients, thereby contributing to an unfavorable prognosis. Conversely, individuals in the lobectomy group may have refrained from receiving adjuvant chemotherapy or treatment upon recurrence, or they might have received diminished chemotherapy due to the extent of postoperative invasion. Although this study did not identify any discrepancies in performance status (PS) or respiratory function, it is possible that the lobectomy group had lower activities of daily living (ADL) after pancreatic cancer surgery. Therefore, it is important to carefully consider the surgical approach.

This study had several limitations. First, the relatively small sample size limits the statistical power of our findings. Second, there may have been differences in patient selection across institutions. While the indications for lung nodules were consistent with those outlined in the Materials and Methods from the thoracic surgeon’s perspective, selection bias may have occurred prior to referral to the thoracic surgery department, as decisions were initially made by the physician managing the primary tumor. Third, this was a retrospective study rather than a prospective trial. Fourth, genetic analyses were not performed on individual metastatic tumors. Lung metastases and primary lung adenocarcinoma may be difficult to distinguish, especially IMA. Nonetheless, a parallel trend was noted in the analysis that omitted IMA, indicating that its exclusion did not exert a notable influence on the outcome of this study. Further large-scale studies in this area are necessary. Our study is significant because only a few studies have been conducted on the resection of primary lung cancer following pancreatic cancer surgery.

In contrast to conventional lung cancer, the poor prognostic outcomes observed in this study raise questions regarding the superiority of surgery over systemic or stereotactic body radiation therapy. Recent literature has highlighted the efficacy of stereotactic body radiation therapy in the management of primary lung cancer [16]. Consequently, the findings of this study imply that certain patients with primary lung cancer after pancreatic cancer may derive benefits from surgery. Nevertheless, further investigations are warranted to ascertain the optimal treatment modality for these individuals. The presence of long-term survivors and the complexity of making a preoperative diagnosis based on imaging findings suggest that surgery, when tolerable by the patient, maybe a viable option.

## Conclusions

This retrospective analysis showed that some patients achieved long-term survival with primary lung cancer after pancreatic cancer surgery. However, the prognosis appeared to be worse than that of typical lung cancer cases, especially in patients who underwent lobectomy. Therefore, it is important to carefully consider surgical indications and procedures.
